# Metabolism, pharmacokinetics, and bioavailability of ZB716, a Steroidal Selective Estrogen Receptor Downregulator (SERD)

**DOI:** 10.18632/oncotarget.21808

**Published:** 2017-10-10

**Authors:** Changde Zhang, Shanchun Guo, Lin Yang, Jiawang Liu, Shilong Zheng, Qiu Zhong, Qiang Zhang, Guangdi Wang

**Affiliations:** ^1^ Department of Chemistry, Xavier University of Louisiana, New Orleans, LA 70125, USA; ^2^ RCMI Cancer Research Center, Xavier University of Louisiana, New Orleans, LA 70125, USA; ^3^ Chongqing Medical and Pharmaceutical College, Chongqing, 401331, China

**Keywords:** ZB716 metabolism, oral SERD, pharmacokinetics, sulfation, glucuronidation

## Abstract

ZB716 is a selective estrogen receptor downregulator (SERD) with excellent oral bioavailability and superior efficacy. In this study, we investigate the *in vitro* and *in vivo* metabolism and the pharmacokinetics of ZB716 by incubation with liver microsomes, liver cytosol, and by orally dosing rodents. Metabolic products were identified and quantified by a combination of liquid chromatography and tandem +mass spectrometry. The metabolic profile of ZB716 showed fulvestrant and ZB716-sulfone as the two major oxidative metabolites. ZB716 also underwent some degree of sulfation and glucuronidation *in vitro*. The major oxidative metabolites of ZB716 were found in rat plasma, feces, and urine samples. No sulfation and glucuronidation metabolites from ZB716 were found in plasma. Limited amounts of sulfate conjugates and glucuronides of ZB716 were detected in feces. The glucuronidation on 3-OH position of fulvestrant was the main metabolite found in urine, suggesting that this specific site of phase 2 metabolism is blocked in ZB716 and formation of glucuronide 3-fulvestrant must be preceded by metabolic transformation of ZB716 to fulvestrant. The pharmacokinetic study of ZB716 showed a half-life (t_1/2_) at 17.03 hour, the area under curve value (AUC) of 1451.82 ng/ml*h, and the maximum plasma concentration (C_max_) at 158.12 ng/mL reached at 2 h after a single dose of 10 mg/kg by oral gavage. Overall this study elucidated important metabolic characteristics of ZB716, an oral SERD that has demonstrated superior bioavailability and efficacy in preclinical studies conducted so far.

## INTRODUCTION

Fulvestrant is the only FDA approved selective estrogen receptor downregulator (SERD) indicated for estrogen receptor (ER) positive, metastatic breast cancer that has progressed upon tamoxifen or aromatase inhibitor (AI) treatment [1–2]. However, the drug is not orally bioavailable, and its high-dose monthly regimen of 500 mg as an intramuscular injection produced limited drug exposure and ER turnover in patients [3–4]. In the second, third, or fourth line setting, the low bioavailability of fulvestrant and its slow action may in particular contribute to limited efficacy because endocrine-resistant tumors require an even higher drug exposure [5–8]. The clinical response rate to fulvestrant as a second line therapy in the metastatic setting remains below 20% [9].

Advances in oral SERDs development have been so far confined to nonsteroidal molecules that bind to the ER, act as ER antagonists, and degrade the estrogen receptor [10]. Indeed, several oral SERD candidates have entered clinical trials since 2014, including GDC-0810 [11], AZD9496 [12], and RAD1901 [13]. While these compounds have shown promising preclinical results, including strong antiestrogenic activity, ER downregulating efficacy comparable to fulvestrant, and favorable pharmacokinetic profiles, their clinical performance has yet to be proven. Notably, the dose chosen for phase II trial of GDC-0810 (NCT02569801), at 600mg per day, is indicative of its high concentration requirement to be therapeutically effective, as demonstrated in the phase I trial [11]. Such high dose requirement may add to its potential gastrointestinal tolerability challenge in subsequent clinical development [14]. Indeed, in April 2017, development of GDC-0810 (brilanestrant) was discontinued by Roche [15–16].

Several reports [17–20] have described attempts to develop orally bioavailable steroidal SERDs. However, no pharmacokinetic data are available and no further progress on pre-clinical studies have been reported since 2010. Indeed, these attempts focused on modifications made primarily to the long alkyl chain to increase polarity and solubility but failed to address the main problem that is responsible for the poor bioavailability of fulvestrant, that is, fulvestrant undergoes rapid and extensive O-glucuronidation [21–22] and O-sulfation [23–24] to form polar metabolites that are inactivated and undergo rapid systemic clearance.

To minimize such metabolic inactivation and clearance that prevent fulvestrant from accessing target tissues, we envisioned a solution in which the 3-OH group of fulvestrant is replaced by a boronic acid group to obtain a novel steroidal oral SERD, ZB716. We have previously succeeded in significantly reducing first pass metabolism of hydroxylated drug molecules using boronic acid derivatives and enhancing their systemic bioavailability in circulation [25–27]. Preclinical studies confirmed that this chemical modification can retain sufficient binding affinity of the steroidal moiety of fulvestrant [27]. We found that ZB716 binds to ER with high affinity and exerts its antiestrogenic effect on ER-expressing breast cancer cells [27]. In both tamoxifen naive and tamoxifen resistant breast cancer cells, ZB716 potently inhibits cell proliferation and effectively degrades the hormone receptor in a dose-dependent manner. In mice, we have shown that ZB716 has far superior oral bioavailability when compared to fulvestrant [27–28], and in two breast cancer xenograft models including a patient derived xenograft (PDX) model, ZB716 has proven to be a more efficacious SERD than fulvestrant in inhibiting tumor growth [28].

**Figure 1:**
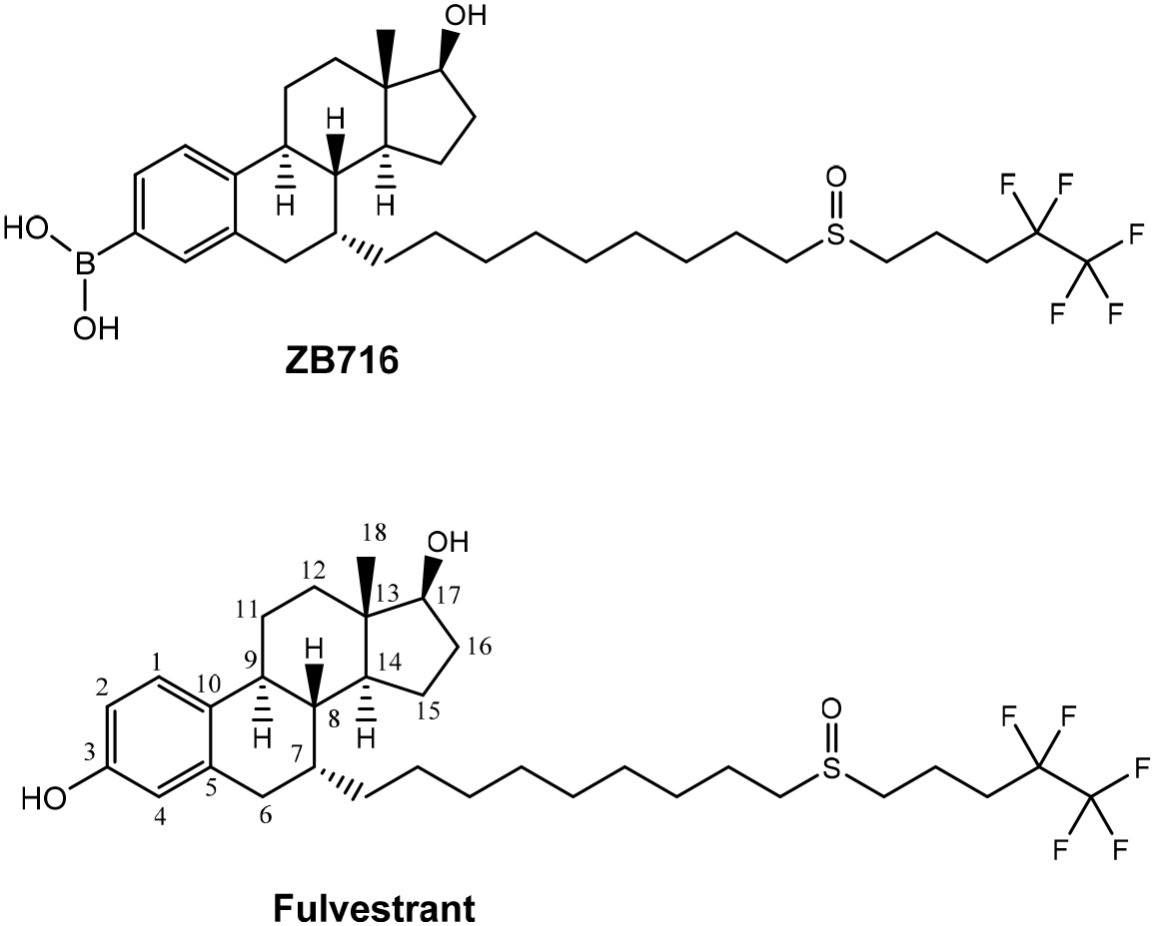
Molecular structures of ZB716 and fulvestrant

The only structural difference between ZB716 and fulvestrant is that the 3-hydroxyl group of fulvestrant is replaced by a boronic acid moiety in ZB716 [27]. The single substitution of -OH with –B(OH)_2_ (Figure 1) is apparently responsible for the vastly improved oral bioavailability while retaining potent biological activities of ZB716 as compared to fulvestrant [27–28]. In mice and rats, oral administration of ZB716 at 10 mg/kg afforded peak plasma concentrations in excess of 100 ng/mL, a level significantly higher than the 20 ng/mL observed for fulvestrant administered at similar dosage via s.c. injection in animals [27–28]. Such superior oral bioavailability of ZB716 was translated to greater efficacy in blocking tumor growth in xenograft models [28]. However, it is not fully understood how the substitution of the phenolic OH group with a boronic acid structure leads to such dramatic enhancement of oral bioavailability as seen in ZB716.

The pharmacokinetics and metabolism of fulvestrant, the sole FDA approved SERD regimen, have been extensively studied [21-24, 29-31]. It has been reported [21] that fulvestrant undergoes rapid glucuronidation catalyzed by UGT1A1, -1A3, -1A4, and -1A8. The predominant site of fulvestrant glucuronidation was found at the 3-hydroxyl position while only 5-10% was fulvestrant-17-glucuronide [21]. Moreover, the high level of UGT1A3 and -1A4 expression in the gastrointestinal tract, two major enzymes responsible for the glucuronidation of fulvestrant at position 3, suggests that fulvestrant may be inactivated both in intestine and in liver [21]. Another metabolic pathway of fulvestrant inactivation and clearance is sulfotransferases mediated sulfate conjugation forming primarily fulvestrant-3-sulfate conjugate [21]. The SULT1 and SULT2 enzymes for fulvestrant sulfation are also expressed in the GI tract where formation of sulfate conjugates may contribute to its overall clearance rate. These observations help explain the poor oral bioavailability of fulvestrant.

In this study we investigate the *in vitro* and *in vivo* metabolism and the pharmacokinetics of ZB716 by incubating liver microsomes and liver cytosols and by orally dosing rodents with ZB716. Liquid chromatography coupled with high resolution tandem mass spectrometry was employed to analyze ZB716 and its related metabolic products in incubation mixtures as well as in rat plasma, urine, and feces samples.

## RESULTS

### *In vitro* oxidative metabolism of ZB716 in rat microsomes

**Figure 2:**
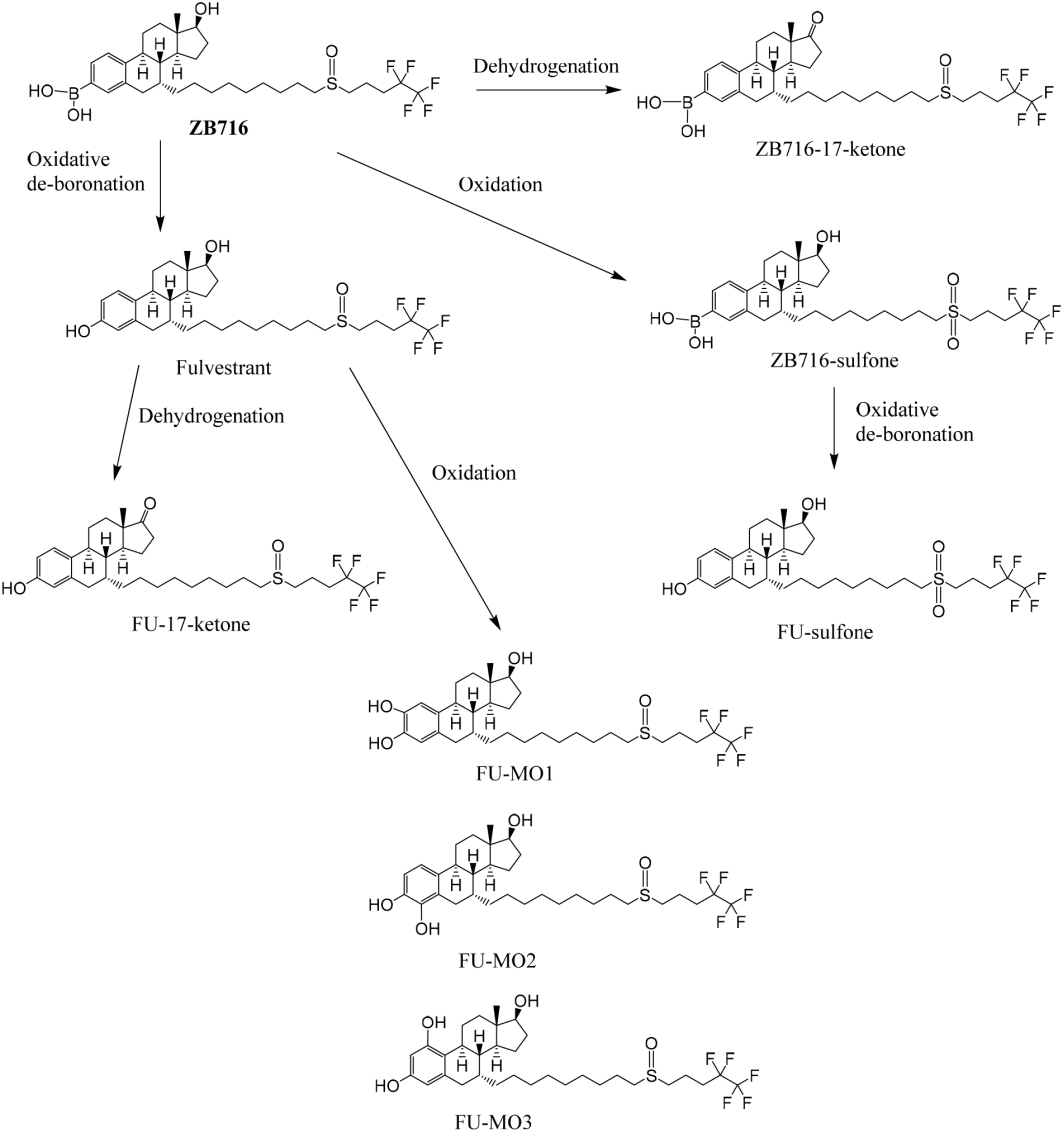
The oxidative metabolic pathways of ZB716 in liver microsomes

NADPH is a cofactor for xenobiotic oxidation reactions. By incubating ZB716 with rat liver microsome in the presence of NADPH for 1 hour, the cytochrome P450 enzymes in the microsomes were allowed to oxidize ZB716 under aerobic conditions. By using a high resolution mass spectrometer, we were able to detect and identify several major metabolites of ZB716 from the incubation mixture. As shown in Figure 2, a total of eight direct or indirect oxidative metabolites of ZB716 were identified. Oxidative de-boronation leads to fulvestrant (FU), which in turn was hydroxylated on each of the available site of the phenyl ring to yield FU-MO1 (2-hydroxy), FU-MO2 (4-hydroxy), and FU-MO3 (1-hydroxyl). Oxidation of the sulfoxide moiety generated ZB716-sulfone and FU-sulfone. Finally, two dehydrogenation products were observed, ZB716-17-ketone, and FU-17-ketone (Figure 2).

**Figure 3:**
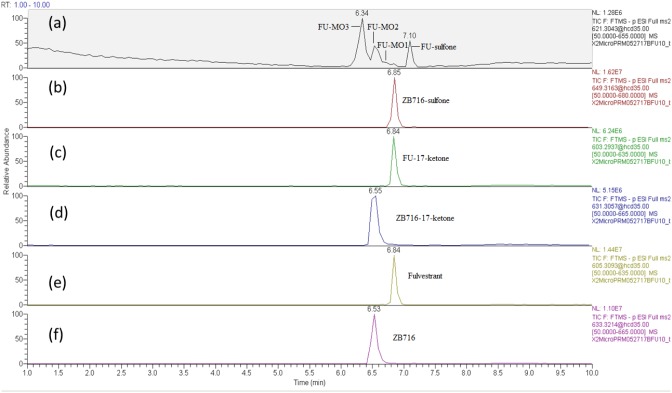
The chromatogram of supernatant of the incubation mixture of ZB716 with rat liver microsomes after 1h incubation

ZB716 and its metabolites are represented by total ion chromatographic peaks at distinct retention times using full scan negative ion mode on the Q-Exactive high resolution mass spectrometer (Figure 3). Their corresponding theoretical mass ions and measured mass ions are listed in Table 1 with mass errors below 1.5 ppm. Unique major fragmentation ions are also given in Table 1, which were obtained with parallel reaction monitoring (PRM) mode on the Q-Exactive instrument. The direct oxidation of ZB716 formed 2 metabolites, fulvestrant and ZB716-sulfone. The oxidative metabolites, FU-MO1, FU-MO2, and FU-MO3 are most likely formed from fulvestrant, rather than from ZB716 followed by de-boronation, because no trace of phenyl-hydroxlated ZB716 metabolites was detected. Although these three metabolites are structural isomers with the identical fragment ion of 443.26 (M-H_2_O-[(CH_2_)_3_CF_2_CF_3_]), the assignment of each isomer was based upon their relative peak area or abundance, which is related to the ease with which hydroxylation may occur on the phenyl ring [32]. Both ZB716-sulfone and FU-sulfone were detected, implying that FU-sulfone could be a product of two possible metabolic pathways, namely, from ZB716-sulfone upon de-boronation, and further oxidation of fulvestrant as the primary metabolite. ZB716-17-ketone was the dehydration product of ZB 716 at 17C position, whereas fulvestrant-17-ketone could be formed by the de-boronation of ZB716-17-ketone and/or by the dehydrogenation of fulvestrant at C17 position.

**Table 1:** Retention time and major fragment ions of ZB716 and its metabolites in oxidation, dehydrogenation, sulfation and glucuronidation analyzed in ESI negative ion mode

Molecular identity	Retention time (min)	[M-H]^-^ Theoretical	[M-H]^-^ Observed	Mass error (ppm)	Major Fragment ions
ZB716	6.53	633.3208	633.3214	0.95	437.26{M-[(CH_2_)_3_CF_2_CF_3_]-2H_2_O}, 445.27, 208.93-[OS(CH_2_)_3_CF_2_CF_3_], 137.02
Fulvestrant	6.84	605.3088	605.3093	0.83	427.26{M-[(CH_2_)_3_CF_2_CF_3_]-O}, 395.29{M-[OS(CH_2_)_3_CF_2_CF_3_]}, 487.26, 445.27
FU-17-ketone	6.84	603.2937	603.2937	0.00	427.26, 395.29, 487.26, 137.02, 168.99
ZB716-17-ketone	6.55	631.3037	631.3057	3.17	437.26, 445.27, 491.29, 137.02
FU-MO1	6.60	621.3037	621.3043	0.97	411.28, 461.26 (M-[(CH_2_)_3_CF_2_CF_3_]), 443.26(M-H_2_O-[(CH_2_)_3_CF_2_CF_3_]), 285.14, 208.93
FU-MO2	6.55	621.3037	621.3043	0.97	443.25(M-H_2_O-[(CH_2_)_3_CF_2_CF_3_]), 152.99, 146.96, 255.23
FU-MO3	6.34	621.3037	621.3043	0.97	443.25(M- H_2_O-[(CH_2_)_3_CF_2_CF_3_]), 208.93, 214.64, 152.99
FU-sulfone	7.10	621.3037	621.3043	0.97	461.27(M-[(CH_2_)_3_CF_2_CF_3_]), 397.31(M-SO[(CH_2_)_3_CF_2_CF_3_], 146.96, 208.93
ZB716-sulfone	6.85	649.3157	649.3163	0.92	427.26, 395.29, 487.26(H_2_O-[(CH_2_)_3_CF_2_CF_3_]), 445.27(M-BO_2_H-[(CH_2_)_3_CF_2_CF_3_]), 208.93, 174.95
ZB716-17-sulfate	5.60	713.2776	713.2782	0.84	553.24{M- [(CH_2_)_3_CF_2_CF_3_]}, 535.23{M- [(CH_2_)_3_CF_2_CF_3_]-H_2_O}, 503.26, 377.12,
FU-3-sulfate	5.97	685.2656	685.2661	0.73	525.23{M- [(CH_2_)_3_CF_2_CF_3_]}, 475.25{M- [(CH_2_)_3_CF_2_CF_3_]+2H}, 427.27, 395.33
FU-17-sulfate	6.54	685.2656	685.2661	0.73	525.23, 475.25, 427.27, 208.93
ZB716-17-glucuronide	5.54	809.3529	809.3535	0.74	113.02, 551.30, 437.26, 649.31{M[(CH_2_)_3_CF_2_CF_3_]}, 599.33{M- [OS(CH_2_)_3_CF_2_CF_3_]-H}
FU-17-glucuronide	6.48	781.3409	781.3414	0.64	527.20{M-CO2-[OS(CH_2_)_3_CF_2_CF_3_]-2H}, 509.19{M-CO2-[OS(CH_2_)_3_CF_2_CF_3_]-H_2_O-2H}, 544.19, 493.21, 144.99
FU-3-glucuronide	5.82	781.3409	781.3414	0.64	621.30{M-[(CH_2_)_3_CF_2_CF_3_]}, 571.32{M-[OS(CH_2_)_3_CF_2_CF_3_]-2H}, 541.29, 445.29, 395

### The formation of ZB716-sulfate conjugates

**Figure 4:**
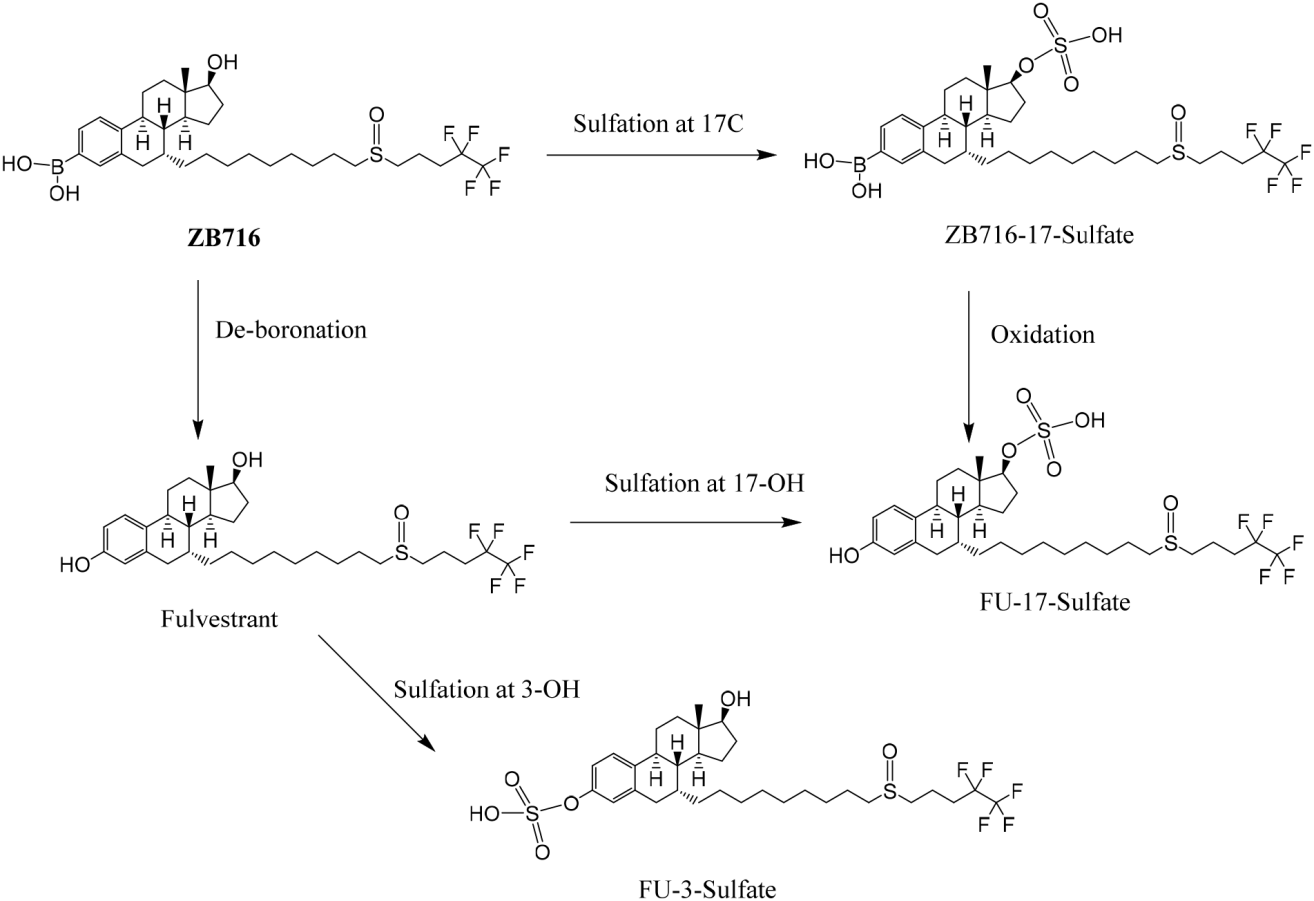
The sulfation metabolism of ZB716 in rat liver cytosols

**Figure 5:**
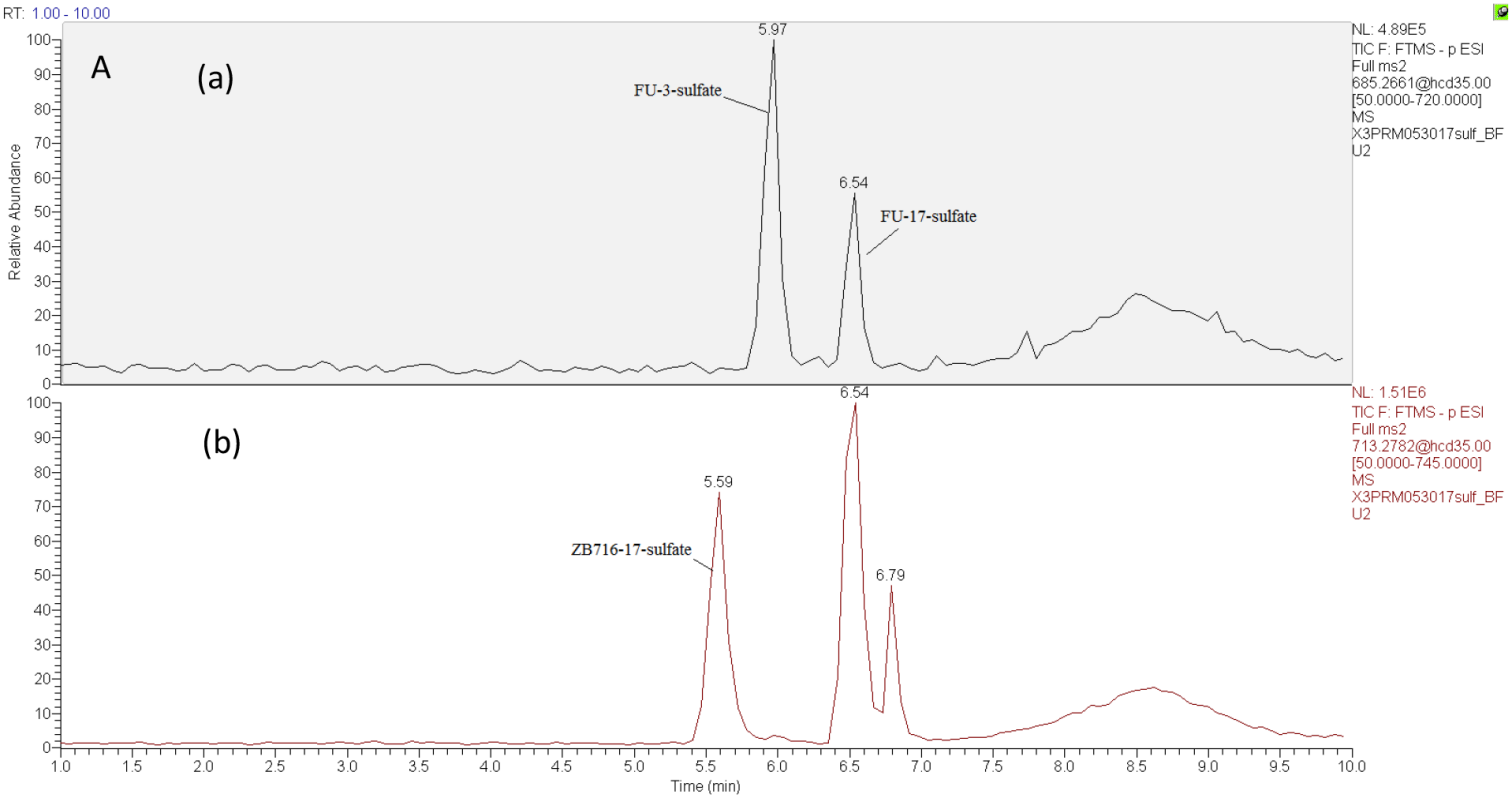

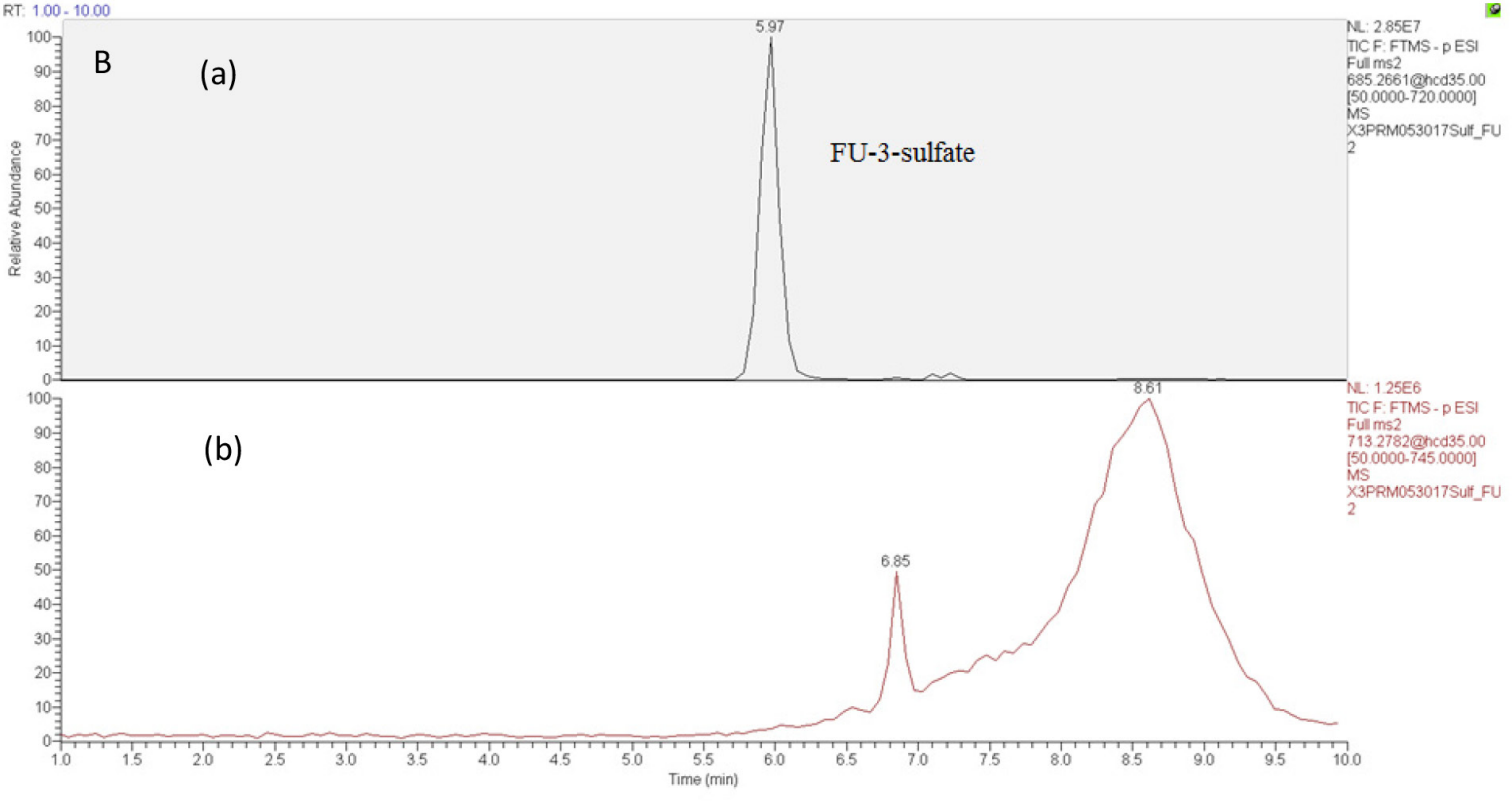
Selected ion chromatograms of liver cytosol incubation mixtures with **(A)** ZB716 or **(B)** fulvestrant.

Sulfate conjugation is enabled by sulfotransferases in the presence of the cofactor 3'-phosphoadenosine-5'-phosphosulfate (PAPS). By incubating ZB716 with rat liver cytosol and PAPS for 30 minutes, we observed three sulfate conjugation products (Figure 4 and Figure 5A). ZB716 formed a sulfate conjugate at the 17-hydroxyl position as determined by its accurate mass filter which yielded a chromatographic peak at 5.6 min. Another sulfate conjugate detected was FU-17-sulfate, which could be the product of deboronation of ZB716-17-sulfate, or that of sulfation of fulvestrant, itself a deboronation metabolite of ZB716. The third sulfate conjugation product was discovered as FU-3-sulfate, also preceded by the formation of fulvestrant from ZB716.

ZB716-17-sulfate showed its unique fragment ions at m/z 553.24 {M-[(CH_2_)_3_CF_2_CF_3_]} and m/z 503.26 {M-2H-SO[(CH_2_)_3_CF_2_CF_3_}. Both FU-17-sulfate and FU-3-sulfate showed its unique 525.23 fragment ion {M-[(CH_2_)_3_CF_2_CF_3_]} and 475.25{M- [(CH_2_)_3_CF_2_CF_3_]+2H} upon collision induced dissociation of their corresponding [M-H]^-^ ions (Table 1). In comparison, incubation of fulvestrant with rat liver cytosol showed that sulfation occurred predominantly at 3-position (Figure 5B) with minimal amount of FU-17-sulfate. This observation indicates that the majority of FU-17-sulfate detected in ZB716 metabolism is likely formed from the de-boronation of ZB716-17-sufate, while the contribution from direct sulfation of fulvestrant may be of negligible importance.

### Glucuronidation of ZB716

**Figure 6:**
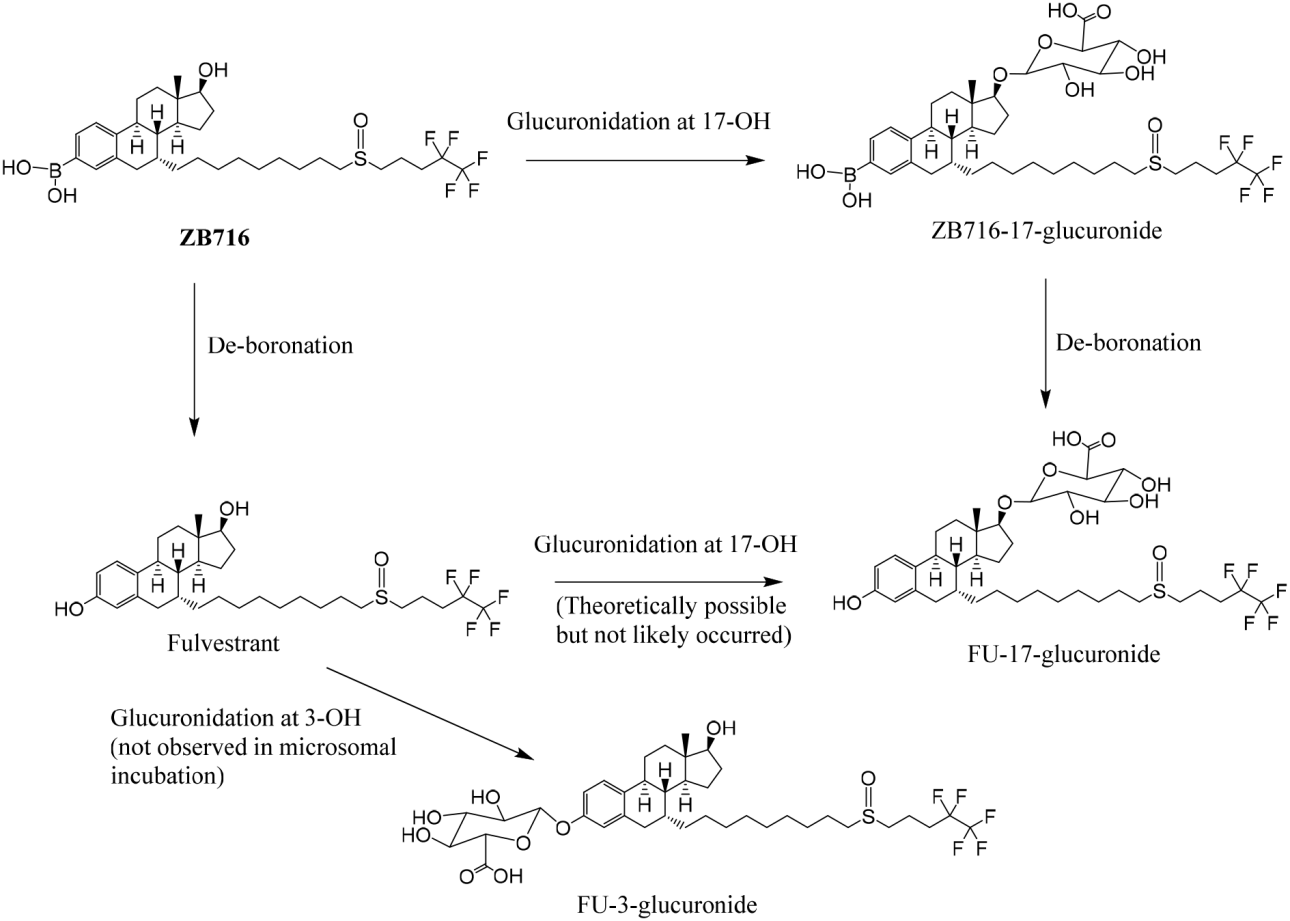
The glucuronidation of ZB716 in liver microsomes and UGT

**Figure 7:**
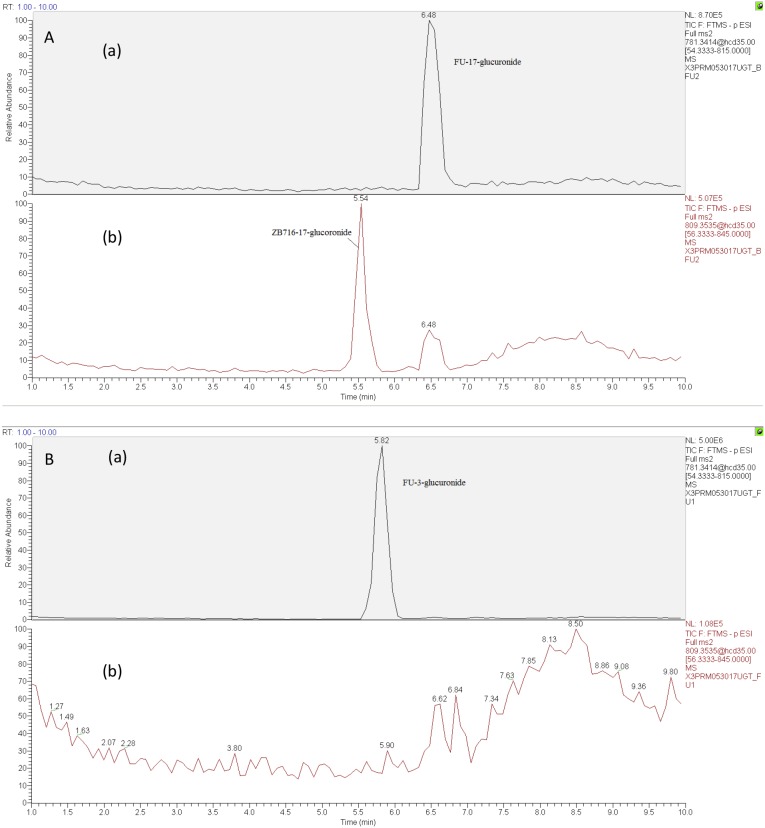
The chromatogram of supernatant of culture solution of ZB716 or FU with liver microsomes and UGT after 1h incubation (glucuronidation) **(A)** ZB716; **(B)** FU.

To investigate the phase 2 metabolism of ZB716 involving glucuronidation, we incubated the compound with liver microsomes supplemented with a UGT reaction mixture Solution A containing uridine diphosphate-glucuronic acid (UDPGA), the cofactor for UGT enzyme. The UGT enzymes in the liver microsomes are allowed to catalyze the conjugation reaction of glucuronic acid with ZB716 to form ZB716-17-glucuronide (Figure 6). Separation by HPLC yielded a peak at 5.45 min that matches the accurate mass of the expected glucuronide (Figure 7A (b)). We also detected FU-17-glucuronide, the conjugate of fulvestrant with glucuronic acid at 17-OH position, which eluted at 6.48 min (Figure 7A (a)).

The detection of ZB716-17-glucuronic acid conjugate was based on its characteristic fragment ions at m/z 649.31{M- [(CH_2_)_3_CF_2_CF_3_]} and m/z 599.33{M- [OS(CH_2_)_3_CF_2_CF_3_]-H} in which the glucuronic acid moiety was retained. The fulvestrant-17-glucuronide was confirmed by its unique fragment ions at m/z 527.20 [M-CO_2_-(OS(CH_2_)_3_CF_2_CF_3_)-2H] and 509.19 [M-CO_2_-(OS(CH_2_)_3_CF_2_CF_3_)-H_2_O-2H] (Table 1). For comparison, we also analyzed the glucuronidation products of fulvestrant after incubation with liver microsomes and UGPGA and confirmed that its glucuronidation occurred primarily at 3-OH (Figure 7B(a)) with negligible amount of FU-17-glucuronic acid conjugate. The majority of FU-17-glucuronic acid conjugate, therefore, was attributable to the oxidative de-boronation of ZB716-17-glucuronide, but not to the direct glucuronidation of fulvestrant, consistent with the observation that incubation of ZB716 with liver microsomes yielded minimal amount of FU-17-glucuronide.

### Metabolism and pharmacokinetics of ZB716 in rat plasma

The same PRM approach was used to identify the metabolites of ZB716 in rat plasma collected at various time points after a single oral dose of 10 mg/kg. The major metabolites of ZB716 identified in rat plasma include fulvestrant, fulvestrant-sulfone, ZB716-sulfone, ZB716-17-ketone, and fulvestrant-17-ketone (Table 2). The pharmacokinetics parameters were calculated using a noncompartment extravascular model (Table 3). Consistent with previously reported pharmacokinetic data in mice [27], ZB716 showed the maximum plasma concentration of 158.12 ng/mL reached at 2 hr after oral administration and the half life time of 17.03 hours. The area under curve (AUC) value of ZB716 in rats was 1451.82 ng/mL*h.

**Table 2:** ZB716 metabolites detected in rat plasma, urine, and feces samples

	Plasma	Feces	Urine
ZB716	+	+	+
FU	+	+	+
FU-17-ketone	+	+	+
ZB716-17-ketone	+	+	+
FU-MO1	-	+	-
FU-MO2	-	+	-
FU-MO3	-	-	-
FU-sulfone	+	+	+
ZB716-sulfone	+	+	-
ZB716-17-sulfate	-	+	-
FU-3-sulfate	-	+	-
FU-17-sulfate (from ZB716-17-sulfate)	-	+	-
ZB716-17-glucuronide	-	-	+
FU-17-glucuronide (from ZB716-17-glucuronide)	-	+	+
FU-3-glucuronide conjugate	-	-	+

**Table 3:** Pharmacokinetic parameters of ZB716 orally administered to rats at 10 mg/kg based on a noncompartment extravascular model

PK Parameter	Value	Unit
**Lambda_z**	0.04	1/h
**t_1/2_**	17.03	h
**T_max_**	2	h
**C_max_**	158.12	ng/mL
**C_last_obs_/C_max_**	0.04	
**AUC _0-t_**	1300.99	ng/mL*h
**AUC _0-inf_obs_**	1451.82	ng/mL*h
**AUC _0-t/0-inf_obs_**	0.90	
**MRT _0-inf_obs_**	18.44	h
**V_z_/F_obs_**	13.97	(mg/kg)/(ng/mL)
**Cl/F_obs_**	0.57	(mg/kg)/(ng/mL)/h

Note: C_last obs_ means the last observed concentration; Cl/F_obs_ means apparent total clearance of the drug from plasma after oral administration; V_z_/F_obs_ means apparent volume of distribution during terminal phase after non-intravenous administration; MRT_0-inf_obs_ means the observed mean residence time from 0→∞; Lambda_z means terminal rate constant.

**Figure 8:**
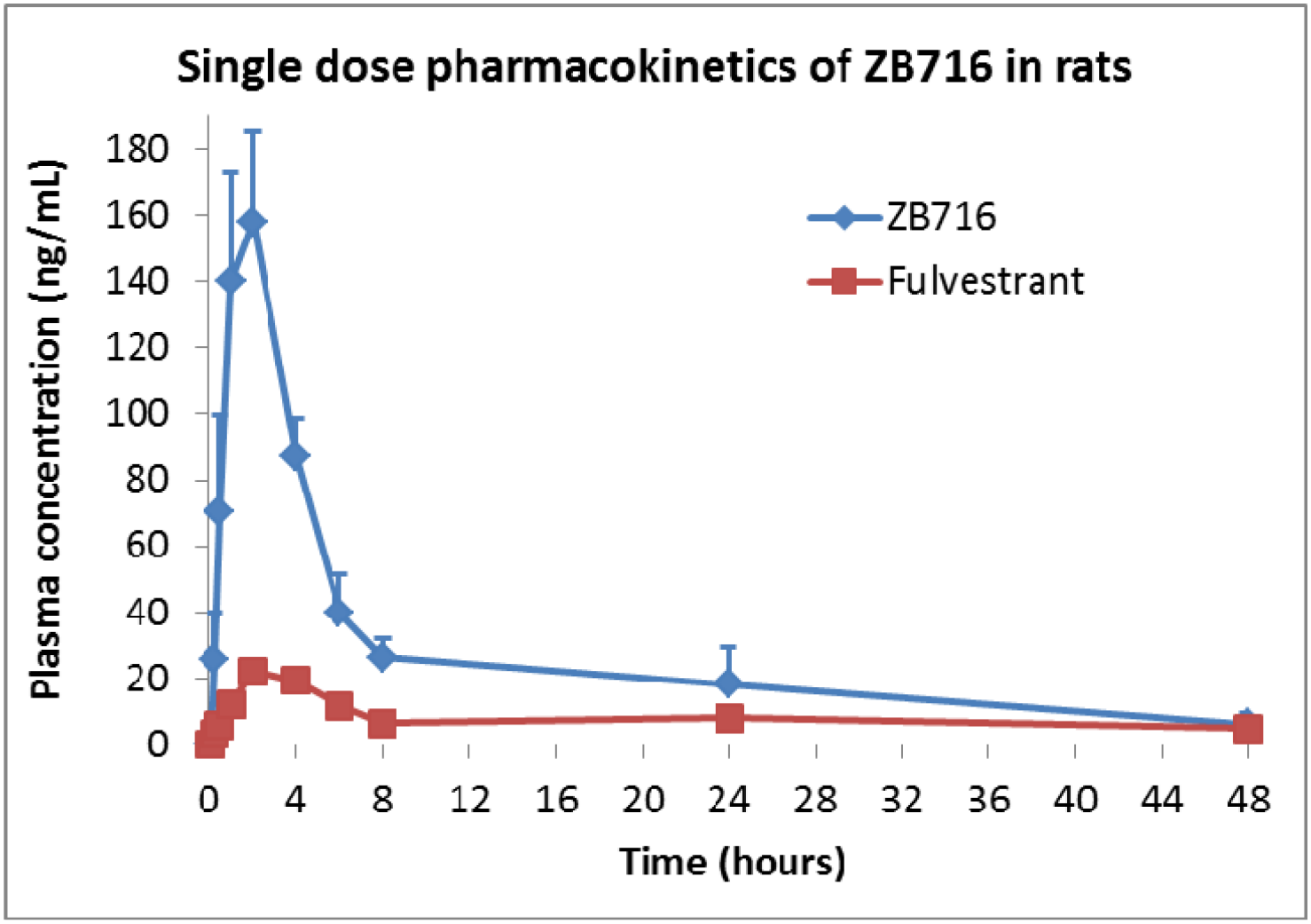
Pharmacokinetics of ZB716 in rat after a single oral dose of 10 mg/kg

The pharmacokinetics curve can be viewed as having five major phases as shown in Figure 8. The 1st phase is where the plasma concentration of ZB716 increased rapidly to from 0 to 1 hour to reach 130 ng/mL. The second phase, from 1 to 2 hour, plasma ZB716 continued to increase, at a slower rate, to peak concentration at 158.12 ng/mL at 2 hr. The third phase is seen from 2 h to 4 h when the plasma concentration of ZB716 decreased quickly to 87.1 ng/mL with 44.9% loss within 2 hours. The fourth phase is from 4 h to 8 h when the plasma concentration of ZB716 decreased at a slightly slower pace to 26.37 ng/mL. The last phase is from 8 h to 48 h in which the plasma concentration of ZB716 decreases from 26.37 ng/mL to 6.91 ng/mL.

### ZB716 and metabolites in rat feces

**Figure 9:**
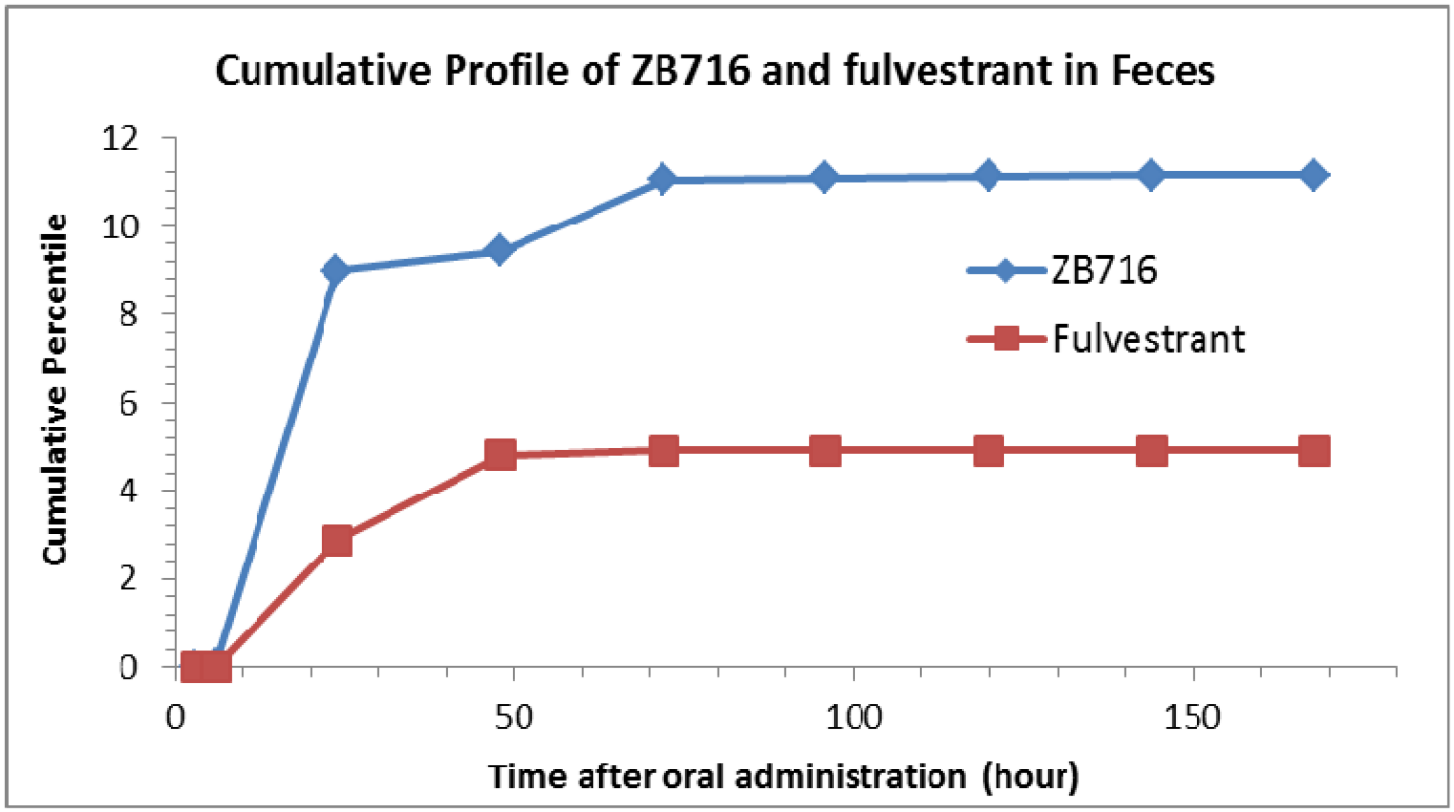
Cumulative profiles of ZB716 and its metabolite fulvestrant in rat feces

The following compounds were detected in rat feces collected at various time points after a single oral dose of ZB716: ZB716, fulvestrant, FU-sulfone, ZB716-sulfone, FU-17-ketone, ZB716-17-ketone, FU-MO1, FU-MO2, ZB716-17-sulfate, FU-3-sulfate, FU-17-sulfate, ZB716-17-glucuronic acid conjugate, and FU-17-glucuronic acid conjugate (Table 2). Due to the lack of standard compounds for most metabolites identified by means of mass spectral information, we were only able to quantitatively determine the amount of two molecules: ZB716 and fulvestrant for which pure standards are available. Low level of ZB716-17-sulfate, FU-17-sulfate and FU-3-sulfate were detected mainly in feces collected at 24 h and 48 h after oral administration but not detectable before 8 h after oral administration. FU-MO1 and FU-MO2 were both detected in rat feces with very low signal. The sulfation and glucuronidation of fulvestrant in feces mainly occurred at the 17-OH position but not at the 3-OH position. No FU-3-glucuronic acid conjugate was detected in all feces samples. After oral administration, 80.7% of total ZB716 present in feces was excreted in 24 hours and another 10.2 % of ZB716 in feces was excreted between 24 h to 72 h (Figure 9). 69.2% fulvestrant was excreted in feces before 72 h. Less than 1% of ZB716 and fulvestrant were measured in feces after 72 hours. About 11.4% ZB716 in total oral dose was excreted through feces.

### ZB716 and its metabolites in rat urine

**Figure 10:**
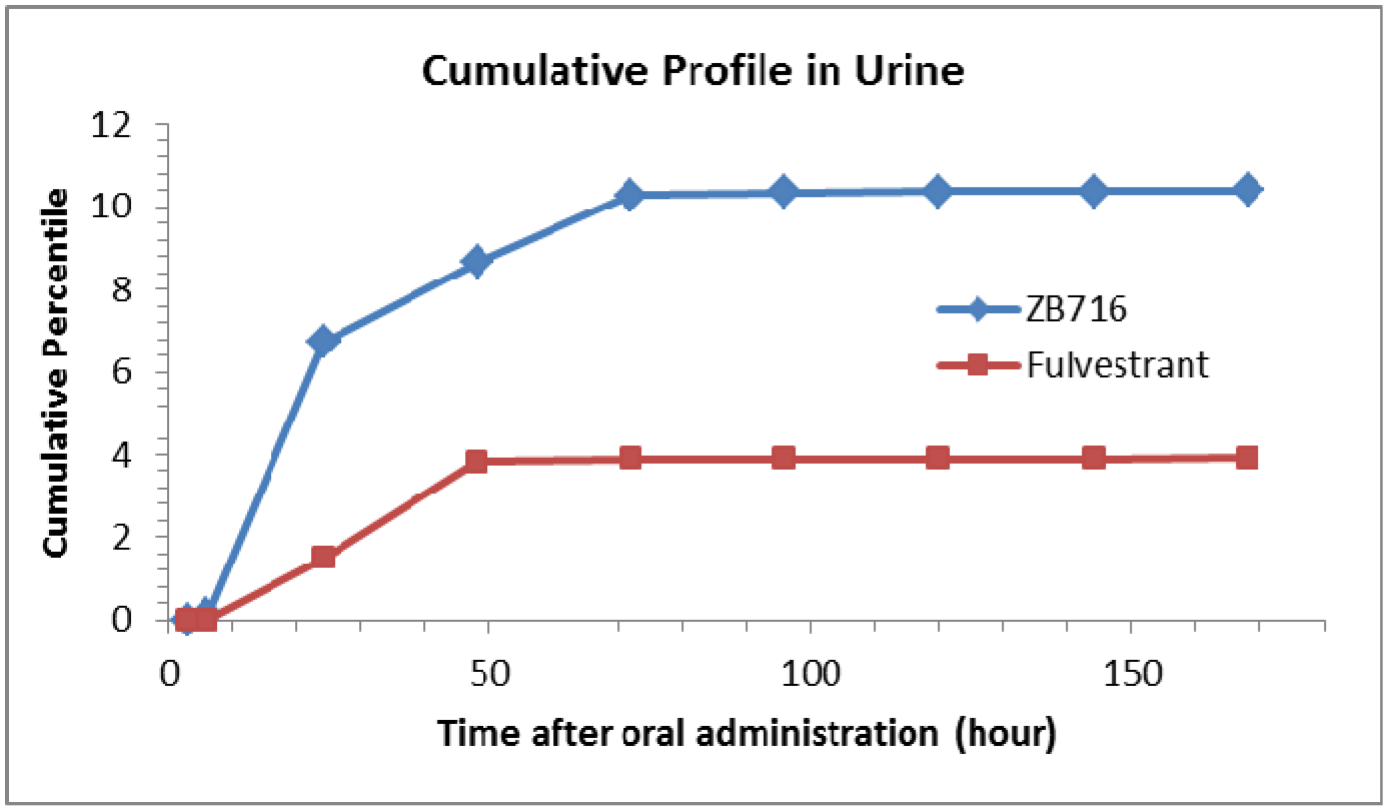
Cumulative profiles of ZB716 and its metabolite fulvestrant in rat urine

Urine samples were subjected to the same HPLC-MS/MS analysis to identify ZB716 and any possible metabolites over a time course post oral administration. In all, we detected six related compounds, including ZB716, fulvestrant, FU-sulfone, ZB716-17-ketone, FU-17-ketone, and FU-3-glucuronic acid conjugate (Table 2). Again, quantitation was only possible with ZB716 and fulvestrant for which standard calibration curves were established. No sulfate conjugate of either ZB716 or fulvestrant was detected in urine. The only glucuronide detected was that of fulvestrant at 3-hydorxyl position. The cumulative profile of ZB716 and fulvestrant (Figure 10) showed that after oral administration, about 63.7% ZB716 in urine was excreted before 24 h and another 34.4% ZB716 in urine was excreted between 24 h to 72 h. 97.6% of all fulvestrant found in urine was excreted in the first 48 hours. Only less than 1% of ZB716 or fulvestrant in urine was accounted for in urinary excretion 72 hours after oral dosage. Nearly 10.4% ZB716 from the oral dose was excreted unchanged through urine.

## DISCUSSIONS

In ZB716, the substitution of fulvestrant 3-OH with a boronic acid has been shown to substantially change its oral bioavailability for the better [27–28]. The metabolic fate of ZB716, therefore, may provide important information on the reason for its enhanced systemic exposure compared to fulvestrant. In the liver microsomal experiment where P450 enzymes are expected to catalyze major oxidative metabolism, the only primary metabolites of ZB716 generated were ZB716-sulfone, ZB716-17-ketone, and fulvestrant. No hydroxylation on the phenyl ring occurred on ZB716. In fact, the only hydroxylated metabolites detected were those of fulvestrant, which were secondary metabolites from oxidation of fulvestrant [29]. Thus, it appears that the 3-boronic acid moiety of ZB716 may effectively block hydroxylation on the phenyl ring, potentially contributing to some degree of reduced metabolism.

Results from metabolic screening in liver microsomes were confirmed in the follow up analysis of rat plasma, urine, and feces samples after oral administration of ZB716. For example, all microsomal metabolites were detected in feces samples except FU-MO3, which was much lower in abundance than FU-MO1 and FU-MO2. In rat plasma, ZB716 represents the major ingredient while a total of five metabolites were detected, including fulvestrant, ZB716-sulfone, ZB716-17-ketone, FU-sulfone, and FU-17-ketone. The hydroxylated metabolites were neither detected in plasma nor in urine samples, indicating that the primary excretion route of these oxidized metabolites is biliary or fecal, but not renal.

The *in vitro* sulfation study of fulvestrant by Edavana et al [24] identified the product of sulfation on fulvestrant 3-position although the authors did not rule out the possibility of existence of fulvestrant 17-sulfate. Our results from incubation of ZB716 with liver cytosols revealed the presence of three sulfate metabolites: ZB716-17-sulfate, FU-3-sulfate, and FU-17-sulfate. The boronic acid moiety at C3 position clearly blocked sulfation at this site, making the 17 position the only site of sulfate conjugation, which may have contributed to significantly reduced first-pass effect compared to fulvestrant. Because fulvestrant is one of the major primary metabolites of ZB716, subsequent formation of fulvestrant 3-sulfate accounts for its presence in the incubation products of liver cytosol and feces. Interestingly, no sulfate conjugates of ZB716 or fulvestrant were detected in plasma and urine, suggesting that the primary route of clearance for sulfate conjugation metabolites is biliary.

Glucuronidation has been recognized as an important metabolic pathway of fulvestrant via formation of mainly FU-3-glucuronic acid conjugate and to a much less extent FU-17-glucuronic acid [21–22]. In contrast, liver microsomal incubation with ZB716 yielded only ZB716-17-glucuronide and FU-17-glucuronide, but not FU-3-glucuronide. This observation clearly demonstrates that the boronic acid in ZB716’s 3-position does not allow glucuronidation at this site and provides further evidence that absence of 3-glucuronidation (dominant in fulvestrant) may significantly reduce first past clearance and contribute to ZB716’s superior oral bioavailability. It is important to note, however, that up to 10-30% of ZB716 can be metabolized to fulvestrant, as observed in mice [27–28] and again in rats in this study, which in turn is subjected to its own metabolic transformation *in vivo*.

The relatively high level of the parent drug ZB716 in urine reflects its high plasma concentration after oral administration. Over 10% of the total unchanged ZB716 was excreted through urine. On the other hand, the cumulative percentage of ZB716 found in feces constitutes about 11% of the total drug administered, compared to many known drugs that are excreted at significantly higher levels in feces in the unchanged form. These results are consistent with the overall metabolic profile of ZB716 which appears to have undergone less extensive phase 1 and phase 2 metabolism when compared with fulvestrant.

In summary, our *in vitro* and *in vivo* metabolism studies show that ZB716 went through 3 routes of 1st phase biotransformation: oxidative de-boronation to fulvestrant, oxidation to ZB716-sulfone, and dehydrogenation to its 17-ketone. These metabolites were found in rat plasma, urine, and feces after oral dosage of ZB716. ZB716 also went through 2^nd^ phase sulfation and glucuronidation, primarily at the 17-position. Both sulfation and glucuronidation activities were weak in the gastrointestinal system with limited amounts of sulfation and glucuronidation metabolites detected in feces. The major glucuronic acid conjugate found in urine was that of FU-3-glucuronide, and to a lesser degree ZB716-17-glucuronide. Considering that FU-3-glucuronide can only be formed from fulvestrant, which is a metabolite of ZB716, it is reasonable to conclude that the glucuronidation of ZB716 is much less facile than fulvestrant.

The metabolic pathways identified in this study revealed an important aspect in the fate of orally administered ZB716: that it is far less susceptible than fulvestrant to inactivation and first pass clearance by the formation of phase II metabolites. The boronic acid moiety in ZB716 that has replaced the 3-OH in fulvestrant effectively prevented the formation of both 3-OH glucuronide and 3-OH sulfate, which are the predominant conjugates observed in fulvestrant metabolism. This may be the main reason for its high oral bioavailability that is clinically valuable for the SERD candidate to achieve high drug exposure and fast therapeutic action.

## MATERIALS AND METHODS

### Chemicals

3'-Phosphoadenosine-5'-phosphosulfate (PAPS) was purchased from R&D Systems. Rat liver microsomes and cytosols were acquired from Gibco CellCite. NADPH solution A, NADPH solution B, UGT Reaction Mix solution A, and UGT Reaction Mix solution B were purchased from Corning Gentest. Tris-HCl buffer (KD Medical) and PBS buffer (Gibco) were used as purchased. Polyethylene glycol 400 was from Sigma-Aldrich Corporation. Ethylenediaminetetraacetic Acid (EDTA) was purchased from Acros Organics through Fisher Scientific. Trans-tamoxifen-13C2, 15N was purchased from ISOTEC through Sigma-Aldrich Corporation; Fulvestrant was from Cayman Chemical Company; ZB716 was synthesized in our laboratory with over 99% purity [27]. Water (HPLC grade), acetonitrile (HPLC grade), methanol, formic acid, dimethyl sulfoxide (DMSO), and other chemicals were from Fisher Scientific.

### Microsomal metabolism of ZB716

NADPH solution A contains NADP+ and Glucose 6-phosphate. NADPH solution B contains Glucose-6-phosphate dehydrogenase, which is necessary for NADPH regeneration. The pre-incubation solution was prepared by adding 30 μL of potassium phosphate buffer (pH 7.4; 10x), 241.5 μL water, 15μL of NADPH solution A, 3 μL of NADPH solution B, and 7.5 μL rat liver microsomes from Corning Gentest into a 1.5 mL microcentrifuge tube. The mixtures were incubated at 37°C for 5 min in an incubator. Then 3 μL of 10 mM ZB716 was added, mixed, and incubated at 37°C for 60 min in the incubator. After incubation, 300 μL MeOH was added to terminate the reaction. The final mixture was then centrifuged at 10,000×g for 4 min at 4°C. The supernatant was analyzed by UHPLC-HRMS (Thermo Q-Exactive).

### Glucuronidation of ZB716 in liver microsomes

The pre-incubation solution was prepared by adding 205.5 μL water, 24 μL of UGT Reaction Mix solution A, 60μL of UGT Reaction Mix solution B, 7.5 μL rat liver microsomes from Corning Gentest into a 1.5 mL microcentrifuge. The mixtures were incubated at 37°C for 5 min in an incubator. Then 3 μL of 10mM ZB716 was added, mixed, and incubated at 37°C for 60 min in the incubator. After incubation, 300 μL MeOH was added to terminate the reaction. The final mixture was then centrifuged at 10,000× g for 4 min at 4 °C. The supernatant was analyzed by the UHPLC-Q-Exactive instrument.

### Sulfation of ZB716 in rat liver cytosols

The pre-incubation solution was prepared by adding 13.5 μL 1 M pH=7.5 Tris-HCl buffer, 247.5 μL water, 6 μL 1 mM 3'-Phosphoadenosine-5'-phosphosulfate (PAPS) from R&D Systems, 30 μL (10 mg/mL) cytosolic protein from Corning Gentest into a 1.5 mL microcentrifuge. The mixtures were incubated at 37°C for 5 min in an incubator. Then 3 μL of 10 mM ZB716 was added, mixed, and incubated at 37°C for 60 min in the incubator. After incubation, 300 μL MeOH was added to terminate the reaction. The final mixture was then -centrifuged at 10,000×g for 4 min at 4 °C. The supernatant was analyzed by the UHPLC-Q-Exactive instrument.

### Identification of ZB716 metabolites in rat plasma, urine, and feces

#### Animals and plasma, urine, and feces sample collection

Female Sprague-Dawley rats, weighing between 350 and 400 g (Charles River Laboratories, Portage, MI), were used for this study on ZB716. Oral gavages containing ZB716 were formulated in 5% dimethyl sulfoxide (DMSO), 40% polyethylene glycol 400, and 55% saline. Rats were housed in metabolic cages for collection of urine and feces samples after oral administration of ZB716 at a dose of 5 or 10 mg/kg. The oral dosage level was based on our previous pharmacology and efficacy studies in mice where it was determined that a dose range of 5 to 20 mg/kg were sufficient to achieve therapeutic effect in mice xenograft tumor models. Urine and feces were collected for the durations of 0-1h, 1-3h, 3-6h, 6-8h, 8-24h, 24-48h, 48-72h, 72-96h, and 96-120 h after drug administration. Blood samples were collected from the lateral tail vein of the rats at 5 min, 15 min, 30 min, 1 h, 3 h, 6 h, 8 h, 24 h, and 48 h time points after drug administration. Rat blood was collected with a capillary into 1.5 mL microcentrifuge tubes containing 0.01 mL of 10 % EDTA anticoagulant. Plasma was then separated from cell pellets by centrifugation in a refrigerated centrifuge at 4 °C and transferred to a separate tube. Plasma samples were frozen at -80 °C until analysis.

### Sample preparation

#### Plasma samples

Plasma samples (100 μL) were extracted using a modified Folch method for lipid extraction. Internal standard (10 μL 0.5 ng/μL trans-tamoxifen-13C2,15N in methanol), methanol (0.5 mL) and chloroform (1 mL) were added to each plasma sample. The mixtures were stored at −20 °C overnight. Samples were sonicated for 10 min and then centrifuged with a Thermo Scientific Heraeus Megafuge16 centrifuge. The top liquid layer was transferred to another test tube. The bottom residual layer was washed with 0.5 mL of chloroform/methanol (2:1), centrifuged, and the top layer was transferred and combined with the previous top layer. The mixture was dried out with nitrogen at room-temperature and re-suspended in 100 μL of HPLC grade methanol for injection.

#### Feces samples

The internal standard of trans-tamoxifen-13C2,15N (0.5 ng/μL in methanol) was added to the feces samples at 10 ng/g feces based on the weight of the feces collected. Feces samples were homogenized with a Power Gen 125 homogenizer (Fisher Scientific) in the mixture of chloroform/methanol (2:1, v:v) and stored at -20 °C overnight. After sonicating for 30 min, the samples were centrifuged with a Heraeus Megafuge16R centrifuge at 3000 RPM. The supernatant was dried with nitrogen gas at room temperature and resuspended with corresponding amount of methanol (200 μL methanol/g feces) for injection on the UHPLC-Q-Exactive instrument.

#### Urine samples

Urine samples were thawed at room temperature and vortaxed. 200 μL of the sample was transferred to a 1.5 mL Eppendorf microcentrifuge tube followed by adding 10 μL of the 0.5 ng/μL trans-tamoxifen-13C2,15N in methanol solution and 200 μL methanol. After Vortaxing and centrifuging, the sample was injected into the UHPLC-Q-Exactive instrument for analysis.

### Analysis of metabolites by UHPLC-High Resolution Mass spectrometry

The Q-Exactive mass spectrometer was coupled to a UHPLC ultimate 3000 (Dionex) using a C-18 column (1.8 μm, 2.1 mm x 50 mm). The gradient started from 30% mobile phase B (acetonitrile with 0.05% formic acid) for 1 min, increased to 100% B in 4.5 min, held for 5 min, then decreased back to 30% B and held for 5 min. Mobile phase A was water with 0.05% formic acid. The flow rate was controlled at 0.3 mL/min. PRM mode was used to obtain the fragment patterns for metabolites with the setting of MS2 resolution of 17500, AGC target of 1e5, maximum IJ of 100 ms, NCE setting of 35. The heated ESI probe setting was set as sheath gas at 30 psi, auxiliary gas at 8 psi, spray voltage at 2500V, capillary temperature at 280°C, S-lens RF level at 50, and auxiliary gas heater temperature at 300°C.

### Quantitative measurement of ZB716 and fulvestrant in plasma, feces, and urine

Concentrated stock solutions of ZB716 and fulvestrant were prepared at 1 mM in methanol. The stock solution of the internal standard (trans-tamoxifen-13C2,15N) was prepared at 1000 ng/μL in methanol. The above stock solutions were kept at -20°C until use. The working solutions of ZB716 and fulvestrant were prepared at 100 μM in methanol. The working solution of the internal standard trans-tamoxifen-13C2, 15N was prepared at 5 ng/μL and 0.5 ng/ μL in methanol, respectively. Working solutions at various concentrations were prepared by diluting their corresponding stock solution with methanol. Calibration standards quality control solutions were prepared over a range of five orders of magnitude. The final calibration solutions for plasma, feces, and urine were prepared by spiking 10 μL of the corresponding stock calibration solution into 0.5 mL extract of blank plasma, or by spiking 20 μL corresponding concentrated calibration solution into 1 mL extract of blank feces, or 1 mL of blank urine/methanol (50:50 v) mixture. The final calibration solutions were prepares at 1, 5, 10, 50, 100, 500, 1000, 5000 nM of ZB716 and fulvestrant and 0.05 ng/μL trans-tamoxifen-13C2,15N, respectively. Quality control samples were prepared by spiking 25 μL of fresh diluted corresponding working solutions into 1 mL extract of blank plasma, or 1 mL extract of blank feces, or 1 mL of blank urine/methanol (50:50 v) mixture. The final quality control samples were prepared in the same way by spiking concentrated quality control solutions into extracts of plasma, feces, or urine samples. The final quality control solutions were prepared at 2.5, 200, 800 nM of ZB716 and fulvestrant and 0.05 ng/μL trans-tamoxifen-13C2, 15N, respectively. The calibration standard curves of ZB716 and fulvestrant for plasma, feces, or urine samples were obtained by separately running their corresponding calibration standard solutions on the UHPLC-MS/MS system.
